# Towards a New Plastination Technique for Moisture Management of Western Red Cedar Without Loss of Strength and with Enhanced Stability

**DOI:** 10.3390/ma18184353

**Published:** 2025-09-17

**Authors:** Olivia H. Margoto, Madisyn M. Szypula, Grant R. Bogyo, Victor Yang, Abbas S. Milani

**Affiliations:** 1 School of Engineering, University of British Columbia, Kelowna, BC V1V 1V7, Canada; olivia.margoto@gmail.com (O.H.M.); madisyn.szypula@ubc.ca (M.M.S.); vy@student.ubc.ca (V.Y.); 2 NetZero Enterprises Inc., Penticton, BC V2A 6C8, Canada; grant@nze.global

**Keywords:** plastination, western red cedar, silicone, durability, moisture management

## Abstract

Amidst environmental concerns regarding the use of petroleum-based materials, wood and wood-based products are among the key players in the pursuit of green construction practices. However, environmental degradation of these materials remains a concern during structural design, particularly for outdoor applications. Borrowed from anatomy to preserve human body parts, this study applies and assesses a technique called ‘plastination’ as a new means for moisture management of Western Red Cedar (WRC). Specifically, the proposed technique includes acetone dehydration of WRC, followed by SS-151 silicone vacuum-assisted impregnation and silicone curing. To evaluate the method’s effectiveness, Micro X-ray Computed Tomography (μCT), Fourier Transform Infrared (FTIR) Spectroscopy, Thermogravimetric Analysis (TGA), and static water contact angle measurements were employed. Tensile testing was also performed to quantify the treatment’s effect on WRC’s mechanical properties under moisture conditioning. μCT confirmed an impregnation depth of 21.5%, while FTIR and TGA results showed reduced moisture retention (3.6 wt%) in plastinated WRC due to the absence of hydroxyl groups. Mechanical testing revealed enhanced deformability in treated samples without compromising tensile strength. Upon moisture conditioning, plastinated WRC retained its tensile properties and showed 59% lower moisture absorption and 15% lower weight as compared to conditioned virgin samples.

## 1. Introduction

Wood and wood-based products have long been a cornerstone of the construction sector, valued for their versatility, aesthetic appeal, and renewable nature. Among the tree species commonly used for lumber, Western Red Cedar (WRC) is popular due to its abundance in North America. WRC is primarily used for traditional lumber and has a secondary use for manufacturing siding, roofing, fences, and engineered wood products [1,2,3]. However, the inherent hydrophilic nature of WRC, similar to other species of wood, makes it susceptible to moisture absorption, microbial decay, and biodegradation caused by fungi such as basidiomycetes, which eventually lead to brown and white rot [4]. These environmental vulnerabilities, combined with WRC’s dimensional instability under humid conditions, significantly affect the material mechanical properties and service life, thereby limiting their long-term performance for modern construction designs concerning weathering [5,6]. To overcome this challenge, growing research efforts have focused on investigating various treatment methods that can preserve wood structures and enhance their durability against moisture absorption. Nevertheless, each method presents advantages and disadvantages, as exemplified below.

Traditional wood preservatives offer some protection but often fall short of providing comprehensive and long-lasting solutions [5]. Adding pesticides or biocides to lumber products is a mechanism commonly used to improve the wood durability by decreasing the likelihood of microorganisms causing decay [7]. Popular options include Copper Chrome Arsenate (CCA), Quaternary Ammonium Compounds (QAC), and Sulfonamides [8]. Copper compounds are among the most popular pesticides/biocides; however, it has been reported that copper-tolerant fungi can still affect wood products’ biodeterioration [4]. Similarly, compounds such as Boric Acid (H_3_BO_3_) suffer from inefficiency in protecting the wood from mould fungi, and they can easily be washed out [4]. In addition, these pesticides and biocides do not enhance the mechanical properties and may even introduce undesirable negative effects.

Wood coatings represent another commonly used approach to modify natural wood, primarily to enhance its resistance to environmental stressors. Numerous reviews have evaluated coatings’ effectiveness in mitigating the effects of moisture and ultraviolet radiation [9,10,11]. In addition to coatings, wood has been functionalized for advanced properties, such as switchable optical and thermal behavior for passive heating and cooling, achieving an average temperature modulation of ~5 °C [12]. Another strategy to enhance wood properties is polymer impregnation, which fills both cell cavities and cell walls [13]. This process typically relies on alternating vacuum and high-pressure cycles, where the vacuum phase removes air from the cells and the pressure phase promotes uniform polymer penetration [13,14]. Although this vacuum–pressure method has been shown to improve mechanical properties, penetration is often limited by fibre dimensions [15,16]. In contrast, studies using only vacuum impregnation have reported success at elevated vacuum levels (–90 kPa), where the reduced pressure was sufficient to eliminate entrapped air and fill open pores [17]. Overall, the limitations of vacuum–pressure methods stem from the complex and heterogeneous structure of wood.

Chemical surface modifications provide another unique means to treat wood by imparting hydrophobicity, which boosts durability and improves bonding between natural fibres and the polymer matrix [18]. For instance, Lin et al. [19] chemically treated wood fibres with tannic acid and Fe^2+^ solution, then blended the fibres with diphenylmethane di-isocyanate to form wood polymer composites, which exhibited improved hygroscopic stability, increased stiffness, and reduced moisture uptake and thickness swelling under high humidity and immersion conditions. Another study by Elamin et al. [20] utilized octadecyl-modified montmorillonite and maleic anhydride-grafted polyethylene octene elastomer as compatibilizers in Low-Density Polyethylene/Chinese fir waste composites, reporting enhanced polymer–fibre interfacial bonding and improved tensile strength. Horst et al. [21] examined the effect of impregnating wood with a 1 wt% resol-type phenolic resin and found notable improvements in the strength and moisture resistance of Polylactic Acid (PLA)/wood composites; however, higher resin concentrations led to a substantial decline in mechanical performance.

Building on conventional chemical modification strategies, combining thermal modification with silicon-based treatments has been shown to further enhance wood hydrophobicity and durability. Kamperidou et al. [22] thermally treated black pine sapwood at 180–200 °C for up to 7 h and subsequently applied AE-APTMOS, an organosilane, with alkyd resin. This approach produced stable Si–O–C bonds between silanes and wood hydroxyl groups, increased silica content within the wood, and markedly improved resistance to water washout, with greater incorporation observed at higher thermal treatment temperatures. Despite these advancements in preserving wood’s environmental performance, many regions have yet to widely adopt these methods. In North America, for instance, the higher cost of chemical treatments compared to conventional biocide-based wood protection has restricted their commercial viability [5]. Regardless, new treatment strategies should aim to balance improved environmental performance with minimizing any decline in mechanical properties [21].

State of the art and objective: Plastination, a technique traditionally used in biological tissue preservation, presents a novel method for enhancing the permeability and durability of natural fibres, as shown in earlier studies by the authors [23,24]. Unlike conventional wood treatments, which typically rely on biocides, surface coatings, bulk impregnation, or chemical treatments [9,13,25], plastination offers a fundamentally different approach by first dehydrating the specimens using acetone. This solvent replaces water within the cells and simultaneously preserves the anatomical architecture once occupied by moisture [24,26].

The technique had been successfully demonstrated on bamboo culms based on acetone dehydration followed by impregnation with a three-part curing silicone system (NCS10, NCS6, NCS3), which was subjected to vacuum pressure and finally curing [23]. Once cured, the silicone preserves the natural fibre by forming a hydrophobic barrier within its microchannels, effectively enhancing moisture resistance by 13% while maintaining structural integrity [23]. Although effective, the plastination method was found to be slow and impractical for industrial-scale applications. To address this, an optimized SS-151 silicone-based plastination technique was developed, offering a 40% faster process while enhancing the bamboo culms’ flexural strength by 60% and retaining their biodegradability [24]. However, this optimized approach did not appear to improve the moisture resistance of the bamboo culms [24].

Building on the above results from recent investigations, this study for the first time aims to investigate the feasibility of plastination for moisture management of Western Red Cedar. Unlike bamboo, WRC is a softwood composed of long, thin cells with microstructural dimensions up to seven times smaller than those of bamboo metaxylem vessels [27,28]. Due to its anatomical structure, WRC presents unique challenges and opportunities for plastination. To evaluate the effectiveness of the plastination process, Micro X-ray Computed Tomography (μCT), Fourier Transform Infrared Spectroscopy (FTIR), Thermogravimetric Analysis (TGA), and static water contact angle measurements were employed. Tensile tests were also performed on both virgin and plastinated specimens, before and after conditioning, to evaluate their resistance to moisture-induced degradation.

## 2. Materials and Methods

### 2.1. Materials

Western Red Cedar lumber supplied by P & E Lumber (Penticton, BC, Canada) was used in this study. To create sample coupons, a piece of WRC lumber (3 in × 3 in) was planed into planks 3 mm thick. Following, dog-bone-shaped test coupons were cut using a Step Craft 600 CNC cutter machine (Menden, DE, Germany). The average air-dry density of WRC samples was measured using a Qualitest MDS300 densimeter (Plantation, FL, United States) and found to be 0.419 g/cm^3^ ± 0.016, and the moisture content was measured in accordance with ASTM D4442—method A and was found to be 6.85% ±0.025 [29].

Acetone (>99.5%, Fisher Canada, Ottawa, ON, Canada) was used for dehydration during the plastination treatment. For forced impregnation, SS-151 silicone from Silicone Solutions (Cuyahoga Falls, OH, United States), a one-part self-levelling fast heat-curing adhesive sealant that cures at 130 °C, was selected. According to the material safety data sheet [30], this silicone consisted of Vinyl Silicone Polymer (35–60%), Dimethylpolysiloxane (20–50%), Methyl Hydrogen Polysiloxane (2.0–6.0%), two trade-secret components (10–20% and 1–5%, respectively), and Chloroplatinum Acid Complex (0.001–0.5%). This impregnator was chosen based on its prior success in bamboo plastination [24], where its low viscosity and straightforward curing process proved advantageous for deep and uniform impregnation.

### 2.2. WRC Plastination Steps

**Figure 1:**
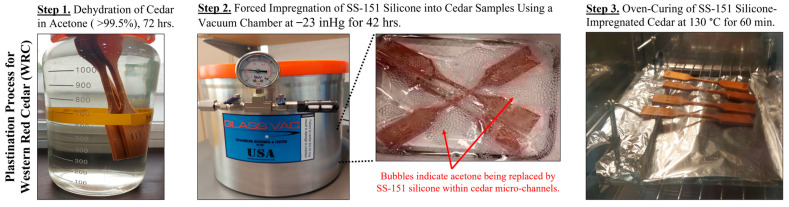
Plastination process applied to the WRC to form WRC/silicone composites.

Following the earlier study by the authors on bamboo [24], the proposed plastination process for WRC consists of three key steps (see also Figure 1):

- Dehydration should occur to replace moisture from WRC with a highly volatile substance, in this case acetone. WRC specimens were fully submerged in acetone for 72 hrs at room temperature (20 ± 2 °C) and stirred every 24 hrs.
- After the completion of dehydration, forced impregnation can be initiated. The dehydrated WRC samples were submersed in SS-151 silicone and placed in a 5-gallon vacuum chamber purchased from McMaster-Carr (Robbinsville, NJ, United States) at a pressure of −23 inHg (−77.9 kPa) for 42 h using a TRIVAC AR4-8 vacuum pump supplied by Leybold GmbH (Cologne, NW, Germany). Under these conditions, acetone vaporizes at room temperature [31], creating a pressure differential that draws silicone into the vacated cellular spaces, allowing it to occupy the voids left behind by the evaporating acetone [23,24]. After vacuum treatment, atmospheric pressure was restored and maintained for an additional 6 hrs to complete the impregnation process.
- Upon forced impregnation, WRC specimens were placed in a 130 °C oven for 60 min to cure SS-151 silicone. Once cured and cooled, the excess silicone was scraped off.

To evaluate the effectiveness of this process, Micro X-ray Computed Tomography (μCT), Fourier Transform Infrared Spectroscopy (FTIR), Thermogravimetric Analysis (TGA), and static water contact angle methods were employed. For μCT, FTIR, and TGA, only one sample per condition was analyzed, as these techniques are highly precise and primarily used to confirm material composition, structure, and impregnation. For contact angle measurements, eight replicates were used. Finally, to access resistance to the moisture-induced degradation, tensile tests were also performed on both virgin and plastinated WRC before and after conditioning, using five replicates per condition. The effect of only the acetone dehydration step on the mechanical properties of WRC was also evaluated, with the results presented in the Supplementary Material.

### 2.3. Characterization

Micro X-ray Computed Tomography: Micro X-ray Computed Tomography (μCT) was performed to visualize the impregnation depth (relative area) of cured SS-151 silicone occupying WRC during plastination, using a MicroXCT 400 scanner by Zeiss (Concord, CA, United States). Plastinated and virgin WRC samples were scanned at a magnification of 4x using a voltage of 40 kV and power of 20 W, resulting in a resolution range of 2–2.5 µm. During the 360-degree rotation scan, a total of 2500 individual images were captured for each sample scan.

Fourier Transform Infrared Spectroscopy: Fourier Transform Infrared-Attenuated Total Reflection (FTIR-ATR) analysis was performed using a Nicolet iS20 device from Thermo Fisher Scientific (Waltham, MA, United States). The FTIR-ATR spectra of the functional groups in the cured SS-151, plastinated WRC, and virgin WRC samples were recorded in the range of 4000 to 500 cm^−1^, with each spectrum consisting of 64 scans and a resolution of 4 cm^−1^.

Thermogravimetric Analysis: Thermogravimetric Analysis (TGA) was conducted to assess the thermal stability of the samples before and after plastination, as well as to estimate the humidity content and silicone percentage in the plastinated sample. Cured SS-151, plastinated cedar, and virgin cedar samples were analyzed using a TGA model Q500 from TA Instruments (New Castle, DE, United States). Samples weighing approximately 10–15 mg were placed in a platinum crucible and subjected to a temperature increase from room temperature to 900 °C at a rate of 10 °C/min, under a 40 mL/min Nitrogen atmosphere.

**Figure 2:**
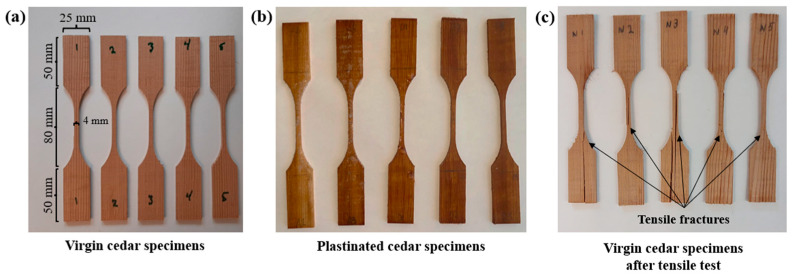
The tensile specimens of (**a**) the virgin and (**b**) the plastinated cedar; (**c**) virgin cedar specimens fractured in tensile mode.

Tensile Testing: Dog-bone-shaped tensile test specimens with a thickness of 3 mm were cut using a Step Craft 600 CNC cutter machine (Menden, NRW, Germany), in accordance with ASTM D4761 [32], with their dimensions shown in Figure 2a. Tensile tests were conducted on five specimens from both the plastinated and virgin WRC groups using an Instron 5969 Universal Tester (Norwood, MA, United States) equipped with a 50 kN load cell and flat grips, at a test speed of 5 mm/min until failure (Figure 2). Tensile testing of acetone-only treated specimens (i.e., only the first step of plastination) was also conducted as additional analysis (see Supplementary Material). All the data obtained from the tensile tests were analyzed using Minitab 18, performing a one-way Analysis of Variance (ANOVA). This analysis specifically aimed to determine, with 95% confidence, whether the three-step plastination treatment had a significant effect on the mechanical properties of the WRC.

Static Water Contact Angle: A Contact Angle Goniometer, manufactured by ramé-hart Instrument Co. (Succasunna, NJ, United States) was employed. A 10 μm droplet of deionized water was dispensed onto the sample surface, and the contact angle was measured using Drop Image Pro software, also manufactured by ramé-hart Instrument Co. (Succasunna, NJ, United States). Measurements were taken at 2 locations on 4 different samples for both virgin cedar and plastinated cedar, resulting in a total of eight measurements per material. ANOVA was applied to statistically assess the effect of the plastination treatment on the contact angle at a 95% confidence level.

Moisture Conditioning: To obtain an insight into the effect of plastination treatment on the environmental durability of the material groups, a moisture absorption test was conducted following ASTM D5229 [33]. This standard includes the non-equilibrium BBFF method, in which specimens are immersed in water for a fixed duration with only initial and final weighing. A four-day soaking period was chosen based on similar tests reported in the literature [23,34], and the observation that most water uptake in softwoods occurred within the first two days [35]. Both plastinated and virgin WRC tensile specimens were weighed and then soaked in separate 1 L water-filled glass bottles at room temperature. After four days, the specimens were removed from the water and wiped free of surface moisture using Kimtech wipes [33]. They were then weighed to determine moisture uptake. Finally, tensile tests were conducted on the conditioned samples to re-evaluate their mechanical performance.

## 3. Results and Discussion

### 3.1. Characterization

**Figure 3:**
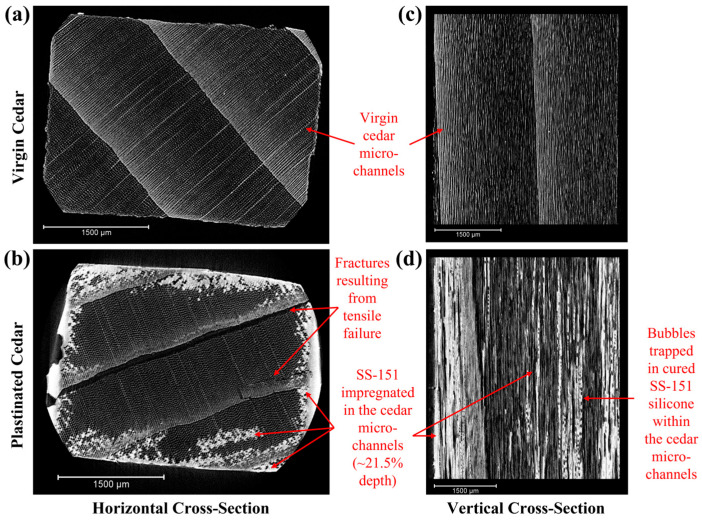
Micro-X-ray images of (**a**) virgin cedar and (**b**) plastinated cedar (horizontal cross section) and of (**c**) virgin cedar and (**d**) plastinated cedar (vertical cross section, i.e., across the microchannels). The horizontal cross section of the plastinated cedar, taken at the center of the dog-bone specimen, shows SS-151 impregnation in the microchannels to a depth of ~21.5%.

Micro X-ray Computed Tomography: The depth of silicone impregnation in plastinated samples was investigated via μCT, with results shown in Figure 3a–d. Since SS-151 silicone has a higher density than virgin cedar, successful impregnation is indicated by a brighter appearance in the X-ray scans (Figure 3a,b). This is because denser materials absorb more X-rays, resulting in increased radiopacity. Figure 3b illustrates that SS-151 silicone is present in the WRC microchannels along the edges of the surfaces; however, minimal amounts of silicone reached the center of the sample. Similarly, in Figure 3d, silicone is present in the microchannels along the edges and in some central channels. Notably, some bubbles are also visible within the cured silicone inside the microchannels. This could be attributed to the incomplete evaporation of acetone during the forced impregnation phase. The application of heat during silicone curing (i.e., step III of the process) may have led to the evaporation of the remaining acetone from step I, resulting in the formation of some bubbles/porosity in the final samples. To mitigate this issue, a controlled drying phase prior to silicone curing could be introduced to ensure more complete solvent removal.

Dhir et al. [23] demonstrated that standard plastination on bamboo yields minimal silicone impregnation (24%), which was optimized to 93% using heat-curing of silicone polymer [24]. However, given the difference in microstructure between bamboo metaxylem (55–216 µm in diameter [27]) and WRC (~30 µm in diameter [28]), the previously optimized procedure in [24] could not be directly applied here to the softwood to achieve the same level of effectiveness [24]. Nevertheless, the preliminary results in Figure 3 indicate that the plastination of WRC is feasible. In this study, the depth of silicone impregnation in WRC samples (Figure 3b) was measured at 21.5%, suggesting partial polymer infiltration. With further optimization, such as extending vacuum duration, modifying solvent systems, or introducing mild pre-treatment steps to enhance cell wall permeability, the impregnation depth may be improved.

**Figure 4:**
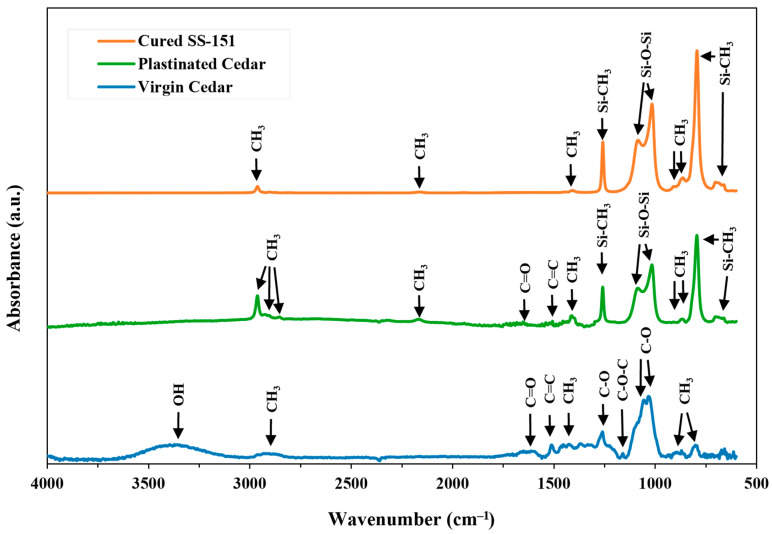
FTIR results for cured SS-151, virgin, and plastinated cedar. The spectra show no evidence of chemical reaction between the cedar functional groups and SS-151 while indicating a reduction in hydroxyl groups associated with moisture content in plastinated cedar compared to virgin cedar.

Fourier Transform Infrared Spectroscopy: To understand the effect of plastination treatment on the chemical structure and functional groups of WRC, FTIR analysis was performed (Figure 4). The presence of silicone in plastinated cedar was confirmed in FTIR analysis through Si–O–Si and Si–CH_3_ bonds (700–1300 cm^−1^), which align with the SS-151 spectrum and are typically associated with silicone [22,36]. Previous studies showed that organosilane-modified wood exhibits chemically stable silicone incorporation, which contributes to hydrophobicity [22]. In our samples, the observed Si–O–Si and Si–CH_3_ bands indicate stable silicone embedding in the wood matrix, although no direct covalent bonding to wood hydroxyls was detected.

Cedar spectra showed wood-specific functional groups, including C–H stretching (2900–2850 cm^−1^), C=O stretching in cellulose, C=C stretching in aromatic rings (1500 cm^−1^), and C–O bonds in pectin or hemicellulose (1266 cm^−1^) [37,38,39]. The 1140 cm^−1^ band is associated with symmetric C–O–C and asymmetric stretching in cellulose and hemicellulose, while the bands in the 1050–1025 cm^−1^ range are attributed to C–O stretching of secondary and primary alcohols in cellulose. Finally, the 900–600 cm^−1^ bands correspond to C–H stretching out of the plane of the aromatic ring [38,39]. The FTIR spectra of plastinated cedar predominantly showed a combination of SS-151 and virgin WRC chemical structures but with a notable distinction. Specifically, the OH stretching band associated with hydroxyl groups caused by moisture content was absent in plastinated samples [38,39]. This absence supports the efficacy of the plastination in reducing the water/moisture content in WRC.

To assess these changes quantitatively, the relative intensities of the OH stretching band (~3400 cm^−1^) and the CH stretching bands (2900–2850 cm^−1^) were analyzed. The OH band corresponds to polar hydroxyl groups that facilitate water sorption, whereas CH vibrations arise from non-polar groups with low water affinity [40]. Virgin WRC exhibited a prominent OH band, resulting in a higher OH/CH ratio (4.5) compared to the plastinated samples (0), where the OH peak was absent. This reduction in the OH/CH ratio reflects the loss of moisture-associated groups and a shift toward a more hydrophobic characteristic of the material, consistent with the observed improvements in moisture resistance in the earlier studies [38,39,40].

**Figure 5:**
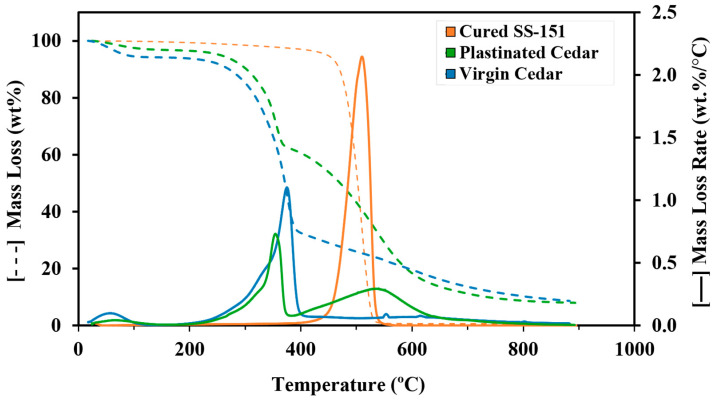
Thermogravimetric analysis (dashed line, mass loss in %) and derivative curves (solid line, mass loss rate in %/°C) of plastinated cedar compared to SS-151 and virgin cedar. The analysis confirms successful SS-151 impregnation (~29 wt% in microchannels and surface coating) and a 3.6% reduction in moisture content compared to the virgin cedar.

Thermogravimetric Analysis: TGA and its Derivative Thermogravimetry (DTG) results for plastinated WRC, along with its two constituent materials (WRC and cured SS-151) are shown in Figure 5, with key data summarized in Table 1. The initial mass loss up to 100 °C is attributed to the moisture removal and indicates that the plastination treatment helped WRC retain 3.6% less moisture compared to the virgin sample, also confirmed by the absence of a hydroxyl group for plastinated cedar samples on FTIR results (Figure 4). Additional evidence from control specimens (e.g., acetone-dehydrated WRC) reinforces the conclusion that acetone dehydration effectively helps in reducing WRC moisture content (see Supplementary Material).

The second degradation stage for the WRC (200–400 °C) is initiated by the decomposition of hemicellulose, the weakest organic component of lignocellulosic materials, and followed by cellulose decomposition, peaking between 355 and 375 °C [41,42]. Silicone was completely degraded at 600 °C, while the virgin and plastinated samples still exhibited weight losses of 9.2% and 9.7%, respectively, in the 600–800 °C range, primarily associated with the remaining degradation of lignin components in the cedar wood [41]. Due to the aromatic ring structure of lignin, its decomposition is slow and occurs at higher temperatures, between 280 and 600 °C, resulting in a significant amount of residue of WRC even beyond 800 °C [41,42].

TGA was also used to estimate the amount of silicone impregnated into the plastinated samples. Within the SS-151 decomposition range (400–600 °C), the mass loss of virgin cedar (42%) and plastinated cedar (13%) was compared to estimate the contributions of each component. Approximately 13% of the 42% mass loss in the plastinated sample is assumed to correspond to the lignocellulosic material of cedar, while the remaining ~29% likely represents SS-151. This TGA estimate of ~29% silicone content agrees reasonably well with the 21.5% impregnation depth determined by μCT. The slightly higher TGA value reflects both the silicone in the microchannels and the surface coating, whereas the micro-CT measurement accounts only for microchannel impregnation.

### 3.2. Effect of Plastination on Mechanical Properties

**Figure 6:**
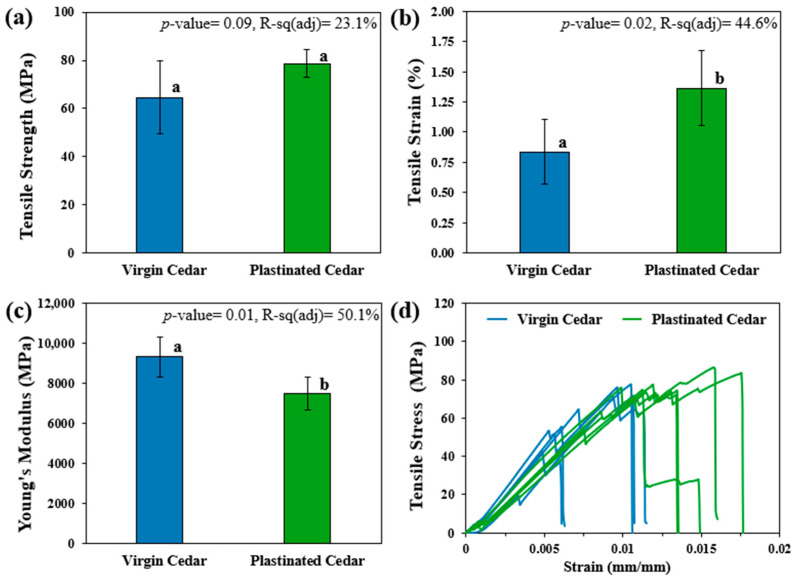
Mechanical properties of the virgin and plastinated (composite) samples: (**a**) tensile strength, (**b**) tensile strain, (**c**) Young’s modulus, and (**d**) stress–strain curves. For graphs (**a**–**c**), the mean value on the bars that do not share the same letter subscript are significantly different, as per ANOVA at 95% confidence.

Tensile testing was conducted to evaluate the impact of plastination on the mechanical properties of Western Red Cedar (WRC), as summarized in Figure 6. Overall, the longitudinal tensile strength of both virgin and plastinated specimens was found to be equal to or greater than the values reported earlier for virgin WRC (45.5 MPa) [43]. Although tensile testing indicated a 21.7% increase in the average strength of plastinated WRC, the improvement was deemed statistically insignificant (*p*-value = 0.09) due to the high variability in virgin cedar (23.6%) compared with low variability in plastinated samples (7.4%). This variability aligns with previous studies on softwood, which report a coefficient of variability in tension of around 25% [43]. It reflects the natural heterogeneity of wood, which arises from differences in specific gravity, fibre arrangement, grain orientation, and the presence of knots or other defects influenced by growth conditions and environmental factors [43]. This suggests that plastination has the potential to enhance the material’s mechanical response stability (repetitiveness) by means of the formed bonding between WRC and silicone matrix in the cell wall structures, while the maximum tensile stress (corresponding to the load-carrying capacity) is maintained [44]. The reduced variability in the mechanical performance of plastinated samples is deemed important as the base natural fibre materials often experience considerable variations in quality, tending to decrease their applicability in high-risk primary structures [45].

Moreover, the results in Figure 6 indicate a statistically significant increase of 63.3% in tensile strain and a 19.8% reduction in the Young’s modulus upon plastination. Similarly, previous bending study [46] on biocide-impregnated western red cedar reported reductions in modulus of elasticity, indicating that decreases in stiffness are observed across different loading modes (flexural and tensile). These observations suggest that plastination increases material flexibility without significantly affecting its maximum tensile strength (Figure 6a). This is hypothesized to result from a higher stretching of the fibre–matrix bond before breaking, which facilitates improved stress distribution between the WRC cell wall structures without compromising the material’s ultimate strength [44].

This increase in flexibility while maintaining tensile strength may offer several functional advantages for plastinated WRC in practical applications. Enhanced deformability can improve impact resistance and reduce the likelihood of brittle failure, particularly under dynamic or cyclic loading conditions; however, further investigation into impact strength, toughness, and resistance to cracking is needed to confirm this hypothesis [47,48,49]. Nevertheless, plastinated WRC’s suitability for load-bearing applications must be evaluated in context-specific scenarios to ensure that reduced stiffness does not compromise structural performance.

### 3.3. Effect of Plastination on Environmental Durability

**Figure 7:**
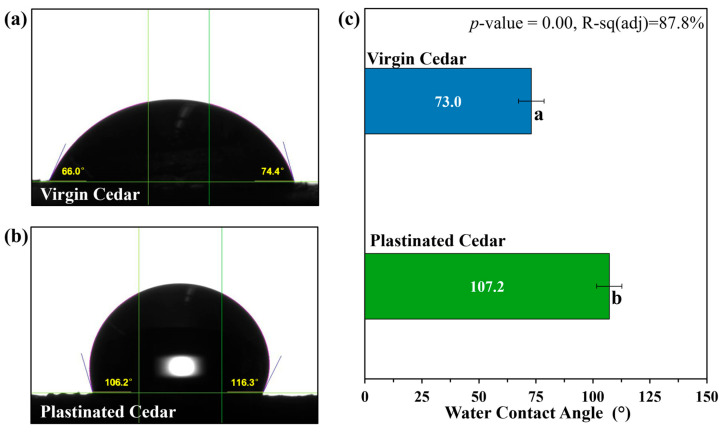
Example of water contact droplet on (**a**) virgin cedar, (**b**) plastinated cedar, and (**c**) overall contact angles of droplets of water on plastinated and virgin cedar. For graph (**c**), the mean value on the bars that do not share the same letter subscript are significantly different, as per ANOVA at 95% confidence.

The effects of plastination on the surface hydrophobicity and wettability are demonstrated by measuring the static contact angle of water on the surface of virgin and plastinated cedar, as seen in Figure 7. Surfaces exhibiting contact angles greater than 90° are classified as hydrophobic [50]. Plastinated cedar was found to have an overall contact angle of 107.2°; meanwhile, virgin cedar was found to have an overall contact angle of 73.9°, suggesting partially wettable surface conditions [51]. This represents an increase of 33.3°, demonstrating that plastination substantially enhances surface hydrophobicity.

The wettability of virgin and plastinated cedar was found to be statistically significant (*p* = 0.00) from each other at a 95% confidence level. This analysis suggests that plastination with SS-151 silicone helped to decrease the wettability of WRC. As SS-151 is classified as a silicone rubber, the addition of methyl groups (CH_3_) could contribute to the hydrophobicity of the plastinated material [52], as also observed in FTIR (Figure 4). Other studies enhancing natural fibre wood materials have found improved hydrophobicity with the use of silicon-based products [53,54,55].

**Figure 8:**
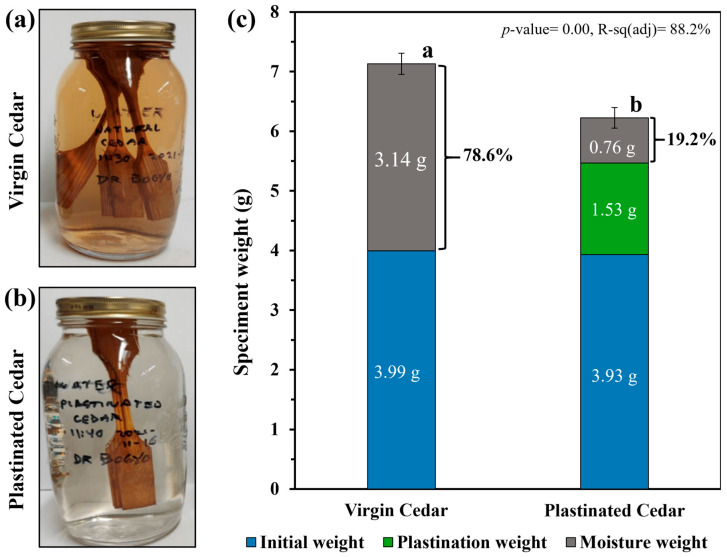
Effect of plastination on cedar after four days of water immersion at room temperature. (**a**) Virgin and (**b**) plastinated composite samples. (**c**) Weight changes due to water absorption, showing that plastination slightly increases initial weight but effectively limits uptake.

The effect of plastination on the moisture resistance of WRC is shown in Figure 8. Upon visual inspection of the samples after four days of water immersion, the solution surrounding virgin WRC exhibited brown pigmentation, indicating leaching of water-soluble extractives abundant in Western Red Cedar [43,56]. In contrast, water around the plastinated sample remained clear (see Figure 8a,b). Similar extractive-related discoloration has been reported for *Pterocarpus marsupium* heartwood after water soaking at room temperature; however, hot-water immersion seemed to reduce water-soluble extractives and prevent brown pigment formation [56]. Pandey et al. [56] explain that in heat-treated wood, leaching could be significantly reduced due to the removal or chemical modification of extractives, which are loosely associated with the cell wall and whose volatile fractions may be lost.

In plastinated WRC, leaching is similarly minimized, primarily because acetone dehydration removes easily leachable extractives while silicone curing locks remaining components in place. However, the effect of acetone on the overall chemical composition of wood, including potential changes to hemicelluloses and extractives, remains unclear. To better understand these effects, future work could employ a coupled TGA-FTIR system on acetone-only treated control samples, along with colorimetric analysis, enabling characterization of extractives in both water and acetone solutions and assessment of any impact on WRC cell wall integrity. In this study, acetone was used for specimen dehydration, following established plastination protocols. While effective at the laboratory scale, the environmental sustainability and economic feasibility of using acetone at larger scales remain concerns due to solvent consumption and cost. Addressing this will require the development of closed-loop solvent recovery systems and a quantitative assessment of cost and recovery efficiency, which we identify as important directions for future work.

As shown in Figure 8c, the weight gain of both virgin and plastinated samples indicates that the plastinated cedar absorbed only 19% water by weight after the moisture conditioning, compared to 78% for virgin WRC. Although the plastination treatment increases the dry weight of the samples due to the infiltration of higher-density silicone into the cedar’s microchannels, plastinated samples remained approximately 15 wt% lighter than virgin western red cedar after moisture conditioning, indicating reduced water uptake and ingress. For comparison, Dhir et al. [23] reported that plastinated bamboo absorbed ~13 wt% less water with 24% impregnation of a three-part curing silicone (NCS10, NCS6, NCS3). In contrast, Osmond [34] observed no significant change in moisture absorption between plastinated and virgin bamboo after four days of water immersion (both ~33 wt%), despite achieving 93% SS-151 impregnation of the bamboo metaxylem vessels under similar conditions. In this study, a 59 wt% reduction in water absorption was achieved with ~21.5% microchannel impregnation, highlighting the superior efficiency of SS-151 silicone in blocking water compared to the standard three-part curing silicone, as well as the improved process adaptability of cedar softwood relative to bamboo given their microstructural differences. Given the significant difference in moisture uptake between the samples, the four-day conditioning period appears sufficient to capture the effect of plastination. Future work examining water uptake kinetics and calculating diffusion coefficients could provide deeper insight into moisture transport mechanisms [35] and enable a more rigorous evaluation of plastinated wood’s moisture resistance.

**Figure 9:**
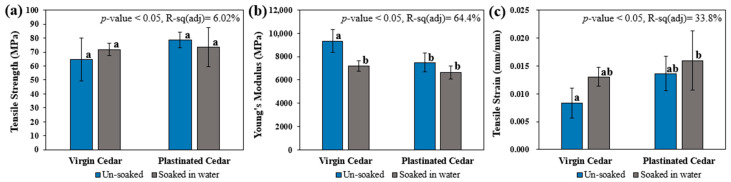
Effect of plastination and moisture on the mechanical properties of WRC: (**a**) tensile strength, (**b**) Young’s modulus, and (**c**) tensile strain. ANOVA was performed to test the overall effect of treatment and moisture at 95% confidence. Bars with mean values that do not share the same letter are significantly different based on pairwise Tukey’s comparisons, with a chosen significance level of *p*-value < 0.05.

The mechanical properties of the virgin and plastinated cedar samples before and after the moisture test can be seen in Figure 9. The results showed that tensile strength was not significantly affected by either plastination (*p*-value = 0.13) or moisture conditioning (*p*-value = 0.84). This can be explained by the failure mechanism of wood loaded parallel to the grain, which occurs primarily within the fibre walls through a combination of cell wall rupture and cell-to-cell slippage, with fibres often tearing obliquely or in a spiral pattern. There is minimal separation along the fibre walls and almost no visible deformation prior to failure. Because the strong cellulose fibres carry the tensile load rather than the weaker interfaces between cells, replacing water with silicone in the cell walls does not appreciably alter the tensile strength along the grain, in contrast to other mechanical properties that are more sensitive to moisture or impregnation [57].

While tensile strength remained largely unaffected, both plastination and moisture conditioning were observed to increase tensile strain and decrease the Young’s modulus of the cedar samples. According to the ANOVA analysis, the change in tensile strain was found to be primarily influenced by the plastination treatment (*p* = 0.02), while the reduction in Young’s modulus (MPa) was more significantly impacted by moisture conditioning (*p* = 0.00), which explained 38% of the variation in the data. An inverse relationship between moisture content and Young’s modulus was observed in the studies [58,59,60,61,62]. This trend aligns with previous findings by Jiang et al., who reported that the tensile Young’s modulus of Chinese fir (softwood) in the longitudinal direction decreased by 24.7% as the moisture content increased [63]. Similarly, in the present study, the Young’s modulus of virgin WCR decreased by 22.9% after moisture conditioning.

It is noteworthy that, unlike the virgin samples, the reduction in the Young’s modulus for the plastinated samples before and after moisture exposure was not statistically significant (Figure 9b). This suggests that the addition of silicone (SS-151) matrix as part of the plastination would make the wood material mechanically more stable during service, which can be particularly advantageous for weathering design of such construction materials. In contrast, other treatments commonly applied to softwoods, such as heat treatment, have been shown to increase dimensional stability and fungal attack but often result in anatomical changes (including cracking of cell walls and enlargement of pits) and reductions of mechanical properties [56,64,65].

Overall, the results highlight the effectiveness and potential of plastination treatment in improving the durability of WRC samples by reducing moisture absorption and making them less prone to degradation. To further enhance this analysis, accelerated weathering testing, specifically focusing on moisture and ultraviolet degradation combined, would provide a more comprehensive evaluation of the environmental durability of plastinated WRC. In addition, increasing the sample size and selecting clear, straight-grained specimens, commonly used in wood mechanics for their relative uniformity (i.e., free of knots, cross grain, checks, and splits), would reduce variability caused by wood defects and provide more robust data on the effects of plastination treatment [43].

## 4. Conclusions

This experimental study evaluated an SS-151-based plastination technique as a novel preservation method for enhancing the environmental durability of WRC while maintaining and stabilizing its mechanical performance. Notably, the treatment resulted in improved moisture resistance, an outcome not previously observed in SS-151-treated bamboo. X-ray Tomography confirmed silicone impregnation into the cedar cell structure, with ~21.5% of cross-sectional microchannels filled. Although deeper penetration may be achievable through further process optimization and/or the use of lower-molecular-weight polymers, current results already demonstrate the significant effectiveness of plastination.

FTIR and TGA analyses showed a reduction in hydroxyl groups and a 3.6% decrease in moisture ingress, indicating improved moisture resistance while maintaining the chemical structure and thermal stability of the WRC. In particular, TGA estimated ~29% silicone content impregnated into the WRC due to plastination, which closely agreed with the impregnation depth (relative area) visualized by μCT. Plastination also led to an increased static contact angle from 73° to 107.2°, highlighting a substantial enhancement in surface hydrophobicity and reinforcing its potential for moisture-resistant applications.

Tensile testing showed that plastination increased deformability by 63.3% without significantly affecting tensile strength, offering the potential to enhance impact resistance and reduce brittle failure in WRC. Most importantly, plastinated WRC samples exhibited notable moisture resistance, absorbing 59 wt% less water and weighing 15 wt% less than virgin WRC after 4 days’ moisture conditioning.

After moisture conditioning, tensile analysis was repeated. While tensile strength remained unaffected, both water-soaked virgin and plastinated samples exhibited increased tensile strain and a decrease in Young’s modulus. Notably, water-soaked plastinated samples did not show a statistically significant reduction in Young’s modulus, unlike their virgin cedar counterparts. This highlights plastination’s role in preserving structural integrity under environmental stress.

Future work should address the cost and sustainability of plastinating lignocellulosic materials by developing closed-loop acetone recovery systems (e.g., re-concentrating the solvent through controlled evaporation of absorbed water) and by exploring the substitution of silicone with bio-based polymers, thereby retaining moisture management performance while reducing reliance on synthetic materials and overall cost. Finally, the effectiveness of the process may also be assessed when the material is in thin (e.g., fibre) form [66], rather than in bulk form, as is commonly the case of reinforcement in advanced composite products.

## 5. Patents

The plastination technique used in this study is based on the patented process (CA3090874) held by G.R.B. of NetZero Enterprises Inc. and was used with permission from the patent holder.

## Figures and Tables

**Table 1:** Thermal degradation profile, including its onset temperature at 5% weight loss, DTG peak temperatures for the main decomposition stages, and residual material.

Sample	Onset _T5%_ (°C)	T_dmax1_ (°C)	T_dmax2_ (°C)	T_dmax3_ (°C)	Residual at 800 °C (%)
Cured SS-151	447.3	-	-	510.0	0.6
Virgin cedar	86.2	57.6	375.0	-	10.3
Plastinated cedar	254.7	66.2	354.4	553.5	8.7

## Data Availability

The original contributions presented in this study are included in the article. Further inquiries can be directed to the corresponding author.

## Supplementary Materials

The following supporting information can be downloaded at: https://www.mdpi.com/article/10.3390/ma18184353/s1, Figure S1: Mechanical properties of virgin and acetone-treated cedar samples: (a) tensile strength, (b) tensile strain, (c) Young’s modulus, and (d) stress–strain curves. For (a)–(c), means with different letter subscripts are significantly different by ANOVA at 95% confidence, *p*-value < 0.05. * indicates that the virgin samples were from a different wood piece than those used in the virgin vs. plastinated tests.

## Abbreviations

The following abbreviations are used in this manuscript:

WRC	Western Red Cedar
μCT	Micro X-ray Computed Tomography
FTIR	Fourier Transform Infrared Spectroscopy
TGA	Thermogravimetry Analysis
CCA	Copper Chrome Arsenate
QAC	Quaternary Ammonium Compounds
PLA	Polylactic Acid
DTG	Derivative Thermogravimetry
