# Supplementary material for: Towards a New Plastination Technique for Moisture Management of Western Red Cedar Without Loss of Strength and with Enhanced Stability
Source: Materials (Basel). 2025 Sep 17;18(18):4353. doi: 10.3390/ma18184353 (PMC12471767; doi:10.3390/ma18184353)
Supplement: Supplementary file 1 [file materials-18-04353-s001.zip › materials-3792922-supplementary.pdf]

Towards a New Plastination Technique for Moisture Management of Western Red Cedar Without Loss of Strength and with Enhanced Stability

Olivia H. Margoto [1], Madisyn M. Szypula [1], Grant R. Bogyo [2], Victor Yang [1] and Abbas S. Milani [1,*]

[1] School of Engineering, University of British Columbia, Kelowna, BC V1V 1V7, Canada; olivia.margoto@gmail.com (O.H.M.); madisyn.szypula@ubc.ca (M.M.S.); vy@student.ubc.ca (V.Y.)
[2] NetZero Enterprises Inc., Penticton, BC V2A 6C8, Canada; grant@nze.global
* Correspondence: abbas.milani@ubc.ca; Tel.: +1-250-8079652

Supplementary Material

S1. Effect of acetone-only treatment in the mechanical properties of Western Red Cedar

To investigate the effect of acetone dehydration on the mechanical properties of Western Red Cedar (WRC), cedar specimens were immersed in acetone for three days following Step I of the plastination process described in Section 2.2. Subsequently, both virgin and acetone-treated specimens were tested in tension according to ASTM D4761, as described in Section 2.3. It is important to note that while the specimens used here were cut from a single piece of cedar, this piece differed from the one used in the virgin vs. plastinated tests. To account for variability between different pieces of wood, virgin tensile tests were repeated using specimens from the same piece as the acetone-treated samples, enabling a direct assessment of the effect of acetone treatment.

Figure S1 shows the tensile strength, tensile strain, and Young's modulus of WRC specimens, focusing on a more detailed understanding of the mechanical performance of the controls (acetone-only treatment without silicone). Compared with the virgin samples used in the plastination treatment (including silicone), the acetone-only specimens exhibit higher tensile strength and strain but a lower Young's modulus. This ~30% variation is consistent with the typical ~25% variability observed in tensile testing of softwoods and can be attributed to the natural heterogeneity of wood, including fibre arrangement, grain orientation, and defects such as knots [1].

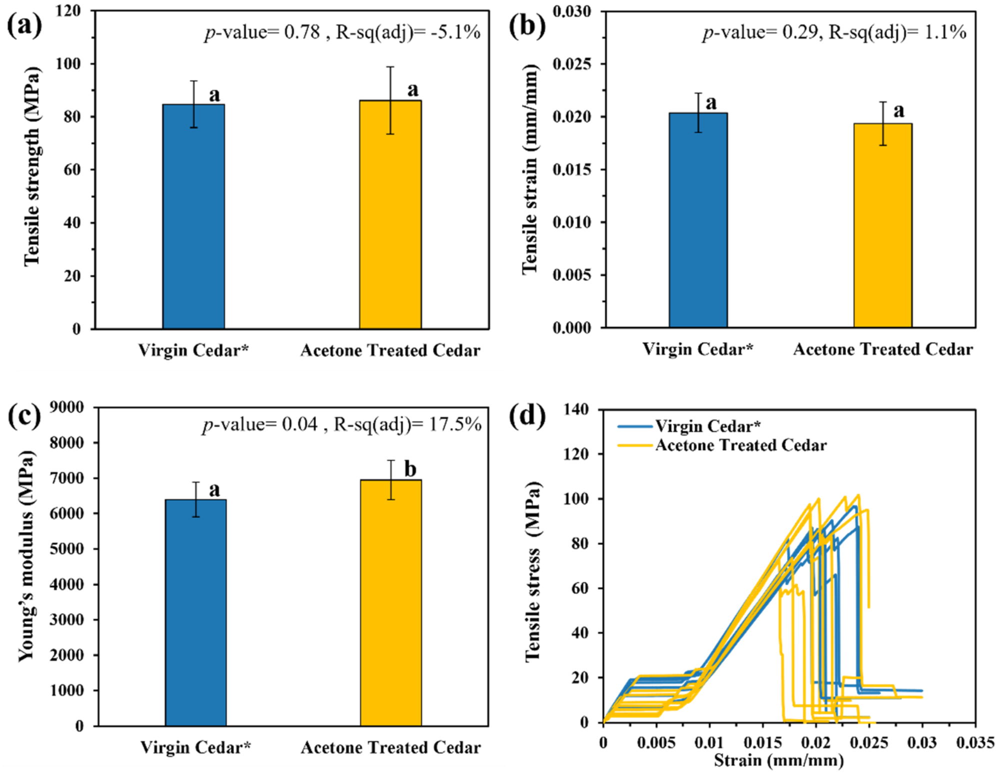

Figure S1: Mechanical properties of virgin and acetone-treated cedar samples: (a) tensile strength, (b) tensile strain, (c) Young's modulus, and (d) stress–strain curves. For (a)–(c), means with different letter subscripts are significantly different by ANOVA at 95% confidence, p-value < 0.05. * indicates that the virgin samples were from a different wood piece than those used in the virgin vs. plastinated tests.

As shown in Figure S1, no significant differences were observed in tensile strength or strain at 95% statistical confidence, indicating that acetone has no statistical effect on these mechanical properties of WRC specimens. A similar trend is reported in the literature, where moisture content was found to have minimal impact on tensile strength parallel to the grain direction [2].

However, Young's modulus appears to be significantly affected, with acetone treatment increasing the average modulus from 6.4 GPa to 6.9 GPa, corresponding to an 8% increase. An inverse relationship between moisture content and Young's modulus was observed in the studies [2–5]. For example, Ozyhar et al. performed uniaxial tensile tests on European beech samples with moisture contents ranging from 7.8% to 16.9% and observed that the tensile modulus decreased as moisture content increased [5].

The increase in Young's modulus observed in cedar following acetone dehydration can be attributed to the behaviour of the wood cell wall. Within the Cellulose Microfibril (CMF) bundles, there exists an intermediate domain that can fluctuate between amorphous-like and crystal-like states depending on moisture content. In acetone-dehydrated samples, it is possible that this intermediate domain shifts toward a more crystal-like configuration, which is mechanically closer to the fully crystalline regions. As a result, the stiffness of the CMF bundle increases, leading to a higher axial Young's modulus. This mechanism likely explains why acetone dehydration enhanced the longitudinal mechanical properties of cedar [2,4]. A study performed by Meng et al. also showed that changing the moisture content from 0 to 6% results in a decrease of about 10.7% of the elastic modulus of pine (*pinus taeda*) wood [6].

References

[1] C.A. Senalik, B. Farber, Mechanical Properties of Wood, in: Wood Handbook: Wood as an Engineering Material, U.S. Department of Agriculture, Forest Service, Forest Products Laboratory, Madison, WI, 2021: pp. 1–46.

[2] Z. Fu, J. Chen, Y. Zhang, F. Xie, Y. Lu, Review on Wood Deformation and Cracking during Moisture Loss, Polymers (Basel) 15 (2023). https://doi.org/10.3390/polym15153295.

[3] M. Báder, R. Németh, Moisture-dependent mechanical properties of longitudinally compressed wood, European Journal of Wood and Wood Products 77 (2019) 1009–1019. https://doi.org/10.1007/s00107-019-01448-1.

[4] H. Yamamoto, Y. Kojima, Properties of cell wall constituents in relation to longitudinal elasticity of wood: Part 1. Formulation of the longitudinal elasticity of an isolated wood fiber, Wood Sci Technol 36 (2002) 55–74. https://doi.org/10.1007/s00226-001-0128-y.

[5] T. Ozyhar, S. Hering, P. Niemz, Moisture-dependent elastic and strength anisotropy of European beech wood in tension, J Mater Sci 47 (2012) 6141–6150. https://doi.org/10.1007/s10853-012-6534-8.

[6] Yujie Meng, Yuzhi Xia, Timothy M. Young, Zhiyong Cai, Siqun Wang, Viscoelasticity of wood cell walls with different moisture content as measured by nanoindentation, AIChE Annual Meeting, Conference Proceedings 5 (2015) 47538–47547. https://doi.org/10.1039/C5RA05822H.
